# A Fast Route Towards Freestanding Single-Crystalline Oxide Thin Films by Using YBa_2_Cu_3_O_7-*x*_ as a Sacrificial Layer

**DOI:** 10.1186/s11671-020-03402-0

**Published:** 2020-08-28

**Authors:** Yao-Wen Chang, Ping-Chun Wu, Jhih-Bang Yi, Yu-Chen Liu, Yi Chou, Yi-Chia Chou, Jan-Chi Yang

**Affiliations:** 1 Department of Physics, National Cheng Kung University, Tainan, 70101 Taiwan; 2 Department of Electrophysics, National Chiao Tung University, Hsinchu, 30010 Taiwan; 3 Center for Quantum Frontiers of Research & Technology (QFort), National Cheng Kung University, Tainan, 70101 Taiwan

**Keywords:** Freestanding, YBa_2_Cu_3_O_7-*x*_ sacrificial layer, Pulsed laser deposition

## Abstract

Researchers have long been seeking multifunctional materials that can be adopted for next-generation nanoelectronics, and which, hopefully, are compatible with current semiconductor processing for further integration. Along this vein, complex oxides have gained numerous attention due to their versatile functionalities. Despite the fact that unbounded potential of complex oxides has been examined over the past years, one of the major challenges lies in the direct integration of these functional oxides onto existing devices or targeted substrates that are inherently incompatible in terms of oxide growth. To fulfill this goal, freestanding processes have been proposed, in which wet etching of inserted sacrificial layers is regarded as one of the most efficient ways to obtain epitaxial high-quality thin films. In this study, we propose using an alternative oxide, YBa_2_Cu_3_O_7_ (YCBO), as a sacrificial layer, which can be easily dissolved in light hydrochloric acid in a more efficient way, while protecting selected complex oxides intact. The high epitaxial quality of the selected complex oxide before and after freestanding process using YBCO as a sacrificial layer is comprehensively studied via a combination of atomic force microscopy, X-ray diffraction, transmission electron microscopy, and electrical transports. This approach enables direct integration of complex oxides with arbitrary substrates and devices and is expected to offer a faster route towards the development of low-dimensional quantum materials.

## Introduction

The semiconductor industry has tremendously influenced modern science and society since the first transistor was built. In order to develop functional materials and devices beyond modern technology, oxide materials are essential due to their versatile physical properties [1, 2]. Over the past decades, complex oxides have captured massive attention due to their versatility, stability, and ease of fabrication [3, 4]. In order to explore hidden properties in materials, high-quality samples are required [5]. Thus, oxide thin film heteroepitaxy appears to be distinctive because it provides high sample quality which is comparable to single crystals. Consequently, the selection of a suitable substrate has become a crucial factor in the growth of high-quality epitaxial films because the structure and properties of the thin films are closely related to interfacial constrain/interaction between the underlying substrate and thin film [6]. The adoption of single crystal substrates for individual materials would significantly affect the performance of complex oxides, due to the lattice mismatch and substrate clamping effect [7, 8]. The requirement of single crystal substrate and constrains resulting from the presence of substrate have essentially made a significant challenge to the integration of epitaxial oxide with silicon-based and practical devices [9]. To conquer the limitation, suitable transfer techniques are required to obtain freestanding thin films which can be further transferred onto desired substrates or existing electronics [10–13]. Hence, the removal of the rigid substrate is a straightforward way to achieve freestanding epitaxial thin films [11–13].

In terms of removal of underlying single crystal substrates while preserving epitaxial films intact, two of the most conventional approaches are laser lift-off and the use of a sacrifice layer. Laser lift-off process was first adopted to transfer GaN from sapphire to silicon [14]. Due to the large bandgap of sapphire substrate, it will not absorb the energy excited by an excimer laser, while the GaN films will absorb most of the pulsed laser energy in a short period of time. With very short laser pulses, GaN would be detached from the sapphire substrate and thus be able to be transferred to silicon or other desired substrates. However, the surface of the transferred film is usually rough due to the bombardment of the excimer laser. Therefore, an annealing process is usually needed after transferred to recover the surface and sample quality [15, 16]. Another common approach is the use of a sacrificial layer inserted between functional oxide film and substrate. The crucial step of this approach is the selection of the sacrificial layer and suitable etching solutions. The sacrificial layer must possess a similar lattice parameter with a desired thin film to enable epitaxial growth without inducing significant amount of defects. In addition to substrate selection, a suitable solution is vital. It shall be noticed that an ideal etching solution only dissolves sacrificial layer and will not do any damage to the desired films. Along this vein, Lu et al. [17] proposed the approach by using Sr_3_Al_2_O_6_ (SAO) as a sacrificial layer to deliver freestanding complex oxide superlattices. The advantage of using SAO is that SAO is water-soluble. It can be easily removed by soaked in pure water. Despite the fact that SAO can be dissolved in neutral water, the large lattice constant of cubic SAO does not match some of functional complex oxides. Besides, the etching rate of SAO is slow, usually taking up to 30 h for etching 20-nm-thick inserted SAO sacrificial layer in most heterostructures. For fast freestanding process, Bakaul et al. [18] carried out epitaxial growth of Pb(Zr_0.2_Ti_0.8_)O_3_ (PZT) thin films on SrTiO_3_ (STO) substrate using (La_*x*_Sr_1-*x*_)MnO_3_ (LSMO) as a sacrificial layer. This bilayer heterostructure was then bathed in potassium iodide/hydrochloric acid (KI/HCl) solution to remove the LSMO layer which can be dissolved by KI/HCI with faster etching rate than PZT. Nevertheless, even though KI/HCI solution etches LSMO in a significantly faster rate, it still causes part of damage on the desired thin films. As a result, seeking for a suitable sacrificial layer with higher selectivity and faster etching rate becomes a critical issue in the development of freestanding thin films.

Here, we propose an efficient freestanding method by adopting YBa_2_Cu_3_O_7-*x*_ (YBCO), which can be easily dissolved in HCl, as alternative sacrificial layer. Utilization of YBCO as a sacrificial layer allows us to separate the desired epitaxial films from the substrates in a much shorter period of time. With a series of structural analysis and functionality verification, we verify that the freestanding process by using YBCO as a sacrificial layer is a universal approach for the fabrication of high-quality complex oxides.

## Methods

### Thin Film Growth

The sample contains two components, the freestanding layer and the buffer layer. Both layers were grown on the STO substrate via pulsed laser deposition using 248-nm KrF excimer laser. The deposition condition may vary from one oxide to another. The YBCO with a thickness of 24 nm was deposited at oxygen pressure of 60 mTorr at 750 °C with a laser power of 200 mJ and a laser repetition rate of 10 Hz, while the LSMO and SrRuO_3_ (SRO) were deposited at oxygen pressure of 100 mTorr at 700 °C with a laser power of 250 mJ and a laser repetition rate of 10 Hz. During growth, reflective high energy electron diffraction was applied to monitor the growth mode and the number of layers of the deposition.

### Etching and Transfer

After deposition, the sample was then spin-coated with poly methyl methacrylate (PMMA), an elastomer layer that provided physical support, keeping it from tearing apart during the following process. The sample was further baked at 120 °C for 5 min to slightly dry up the PMMA on top. The heterostructure covered by PMMA will then be immersed in the 0.6% hydrochloric acid for dissolving the YBCO layer. It takes only a couple of minutes to completely dissolve the sacrificial layer. After the etching process, the heterostructure was dipped in de-ionized water for cleaning. After the cleaning process, we simply pick up the flowing freestanding thin film from the de-ionized water onto desired substrates/devices. Once the film is picked up, it will be baked on a hot plate in order to get rid of the water in the previous stage. Last, acetone was then introduced to remove the support of PMMA.

### Structural Analysis

The crystal structure of the thin film was characterized by synchrotron-based X-ray diffraction techniques at beamline 17A, 17B, and 13A in the National Synchrotron Radiation Research Center, Taiwan.

### STEM Observation

The scanning transmission electron microscopy (STEM) images were taken using JEOL ARM200F equipped with spherical aberration (Cs) corrector at 200 kV accelerating voltage. The semi convergent angle was 25 mrad which formed under 1 Å electron probe, and the semi collection angle of high-angle annular dark-field (HAADF) detector was from 68 to 280 mrad.

### Transport Measurements

The electrical contacts were fabricated in a four-point probe configuration by photolithography process. The contacts were made by Cr (3 nm) and Au (60 nm). Four-point probe is a useful method to measure the resistivity of a sample. Two of the probes are adopted to apply electric current, while the other two probes are used to measure the voltage drop for determing the resistivity. The temperature dependence of resistivity was carried out using Physical Property Measurement System by Quantum Design (PPMS). The sample was firstly heated up to 400 K, and the measurement was performed during the cooling process to reveal the temperature dependence of resistivity.

## Results and Discussion

**Fig. 1:**
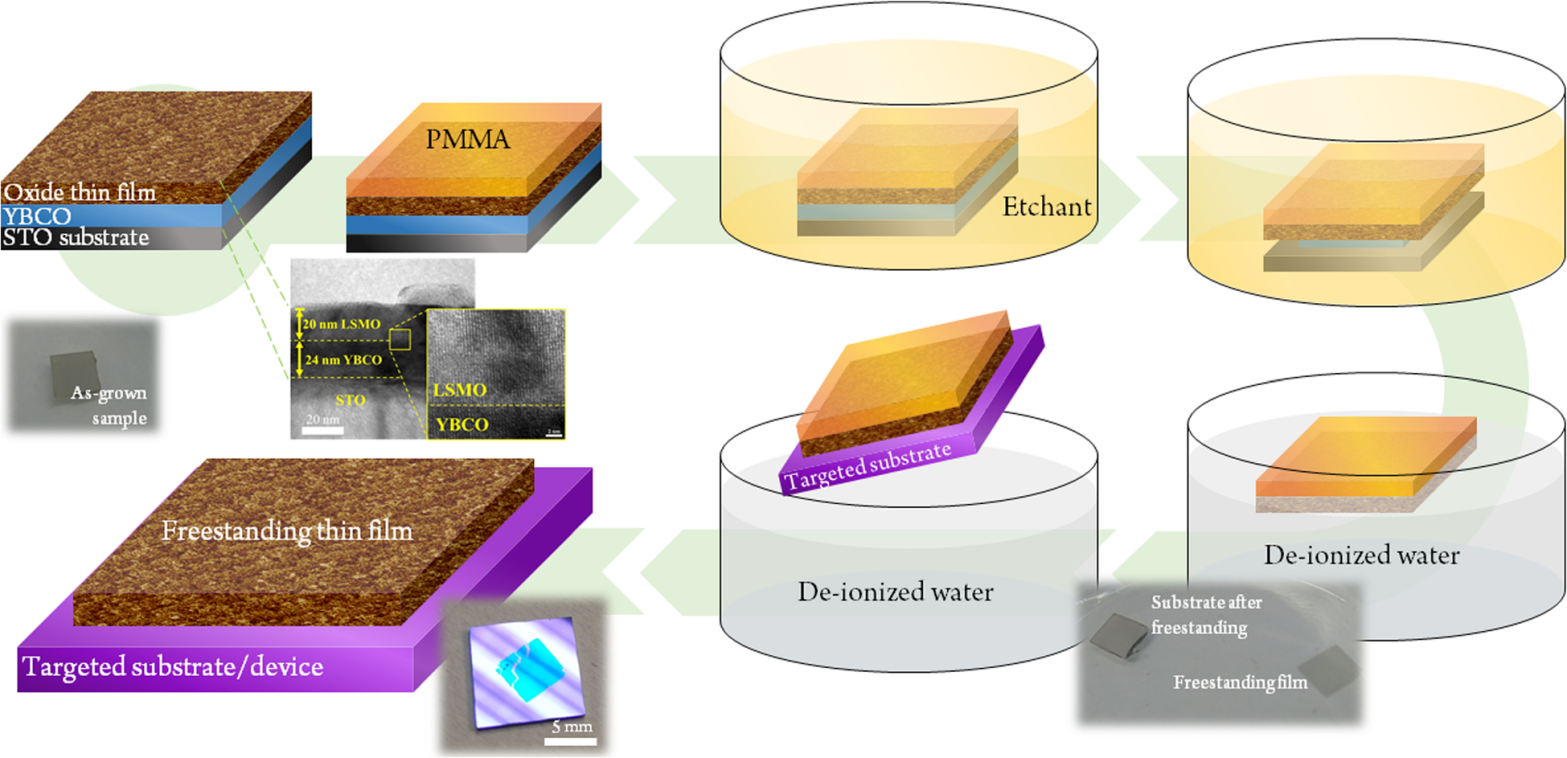
Schematic of the etching process and the transfer of freestanding LSMO layers using YBCO as sacrificial layer.

In this work, an alternative sacrificial layer, YBCO, which can be etched five times faster than LSMO is adopted. YBCO is a high-temperature superconducting material which possesses orthorhombic structure with lattice constants of *a* = 3.82 Å, *b* = 3.89 Å, and *c* = 11.68 Å.^7^ The lattice constants of *a*- and *b*-axes are comparable with most of the complex oxides, allowing high-quality epitaxial growth of desired films on YBCO. The freestanding process using YBCO as the sacrificial layer is illustrated in Fig. 1. To offer a convincing demonstration, LSMO/YBCO heterostructure was firstly deposited on the STO substrate by using pulsed laser deposition, serving as the modeling system. Please be noted that LSMO has been implemented as sacrificial layer for freestanding process in previous studies [18]. After the heterostructure was fabricated, organic PMMA was covered onto the sample to protect the surface and maintain the integrity of LSMO film. The heterostructure was then immersed in light HCl solution to dissolve the YBCO layer. After complete etching of YBCO layer, the sample was put in deionized (DI) water to separate LSMO layer and STO substrate. The LSMO with PMMA could be then transferred to any desired substrates, here, in our study, the silicon wafer. The transferred sample was dipped in acetone for 10 min to remove PMMA. Last, the surface of the freestanding LSMO was cleaned by DI water and isopropanol.

**Fig. 2:**
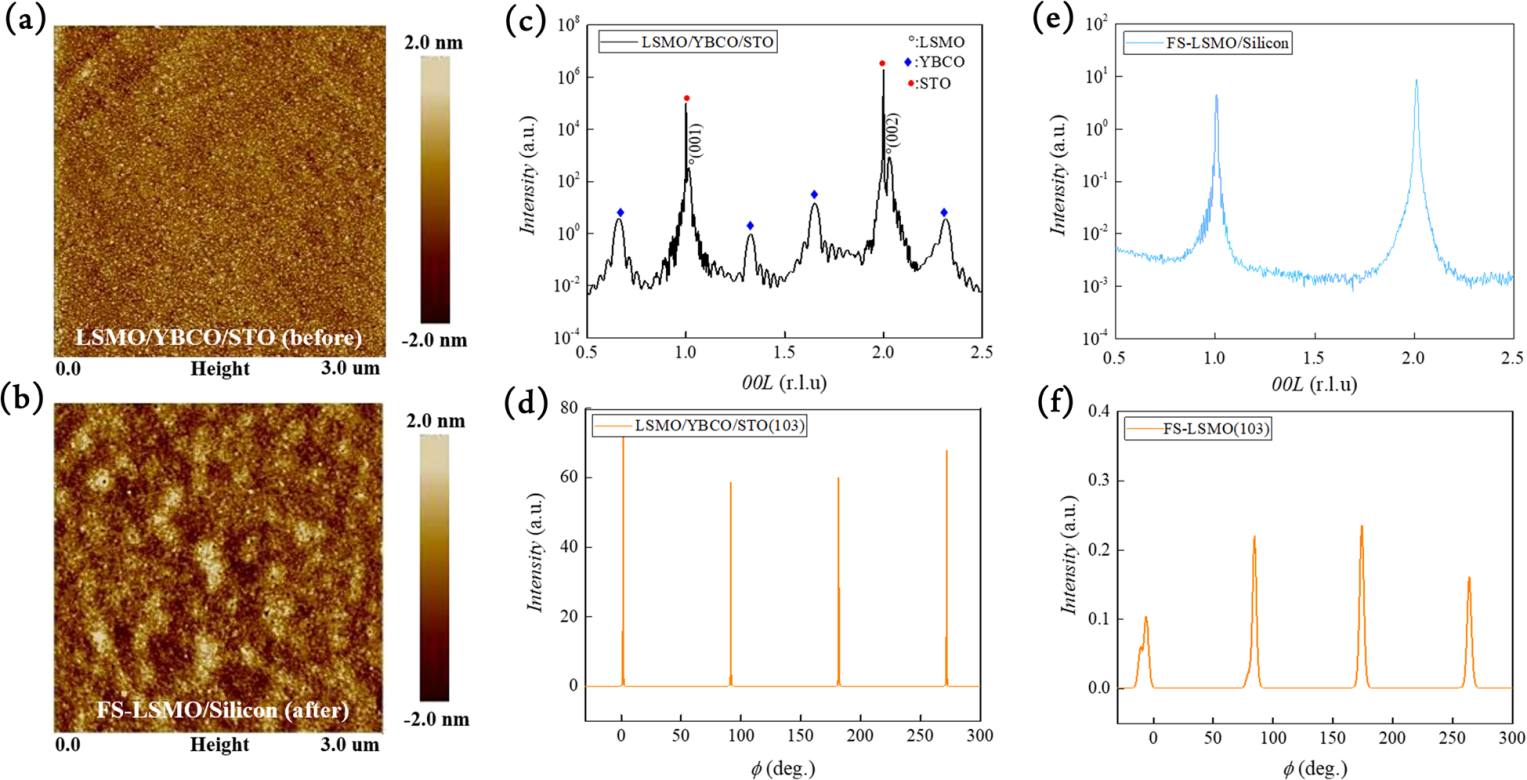
AFM topography images of LSMO **a** before and **b** after freestanding. **c** XRD normal scan of LSMO/YBCO/STO heterostructure. **d** Phi-scan around LSMO (103) plane of as-grown LSMO. **e** XRD normal scan of freestanding LSMO and **f** Phi-scan around freestanding LSMO (103) plane

After transfer, a freestanding LSMO on silicon was obtained. The corresponding surface morphology of LSMO, as-grown and freestanding, are shown in Fig. 2a, b, respectively, indicating flat smooth surface without observable damages on the materials through the freestanding process. To reveal the structural details of LSMO thin films before and after freestanding process, X-ray diffraction (XRD) was employed. The XRD normal scan of as-grown sample, as shown in Fig. 2c, indicates a (001)-oriented LSMO films on YBCO/STO (001). Expect LSMO, YBCO, and STO, there is no secondary phase detected, indicating a pure LSMO feature on YBCO/STO. The calculated *d*-spacing of LSMO extracted from the XRD normal scan is ~ 3.846 Å, which is smaller compared to the bulk value (3.88 Å) [19]. The smaller *c*-axis lattice is expected due to the tensile strain offered by STO substrate (lattice constant = 3.905 Å). Besides, the crystallinity is revealed by the full width at half-maximum (FWHM) (~ 0.051°) from the rocking curve around LSMO (002) (see Supplementary Information Fig. S1a). The phi-scan was employed to confirm the epitaxial relation of LSMO. As shown in Fig. 2d, the reflection of LSMO (103) can be detected every 90°, indicating a 4-fold symmetry of (001)-oriented LSMO film, which is directly correlated to the cubic STO single-crystal substrate. Having established the structural properties of the as-grown LSMO thin film, YBCO layer was then etched by HCl_(aq)_, leaving the LSMO layer floating in the liquid. The freestanding LSMO film was then transferred onto a silicon substrate. A pure (001)-oriented LSMO feature was revealed by XRD normal scan (Fig. 2e), while the phi-scan shows 4-fold symmetry (Fig. 2f). These observations verify there is no structural changes on epitaxial LSMO film after freestanding process. The FWHM of freestanding LSMO layer is also shown in Supplementary Information Fig. S1b.

**Fig. 3:**
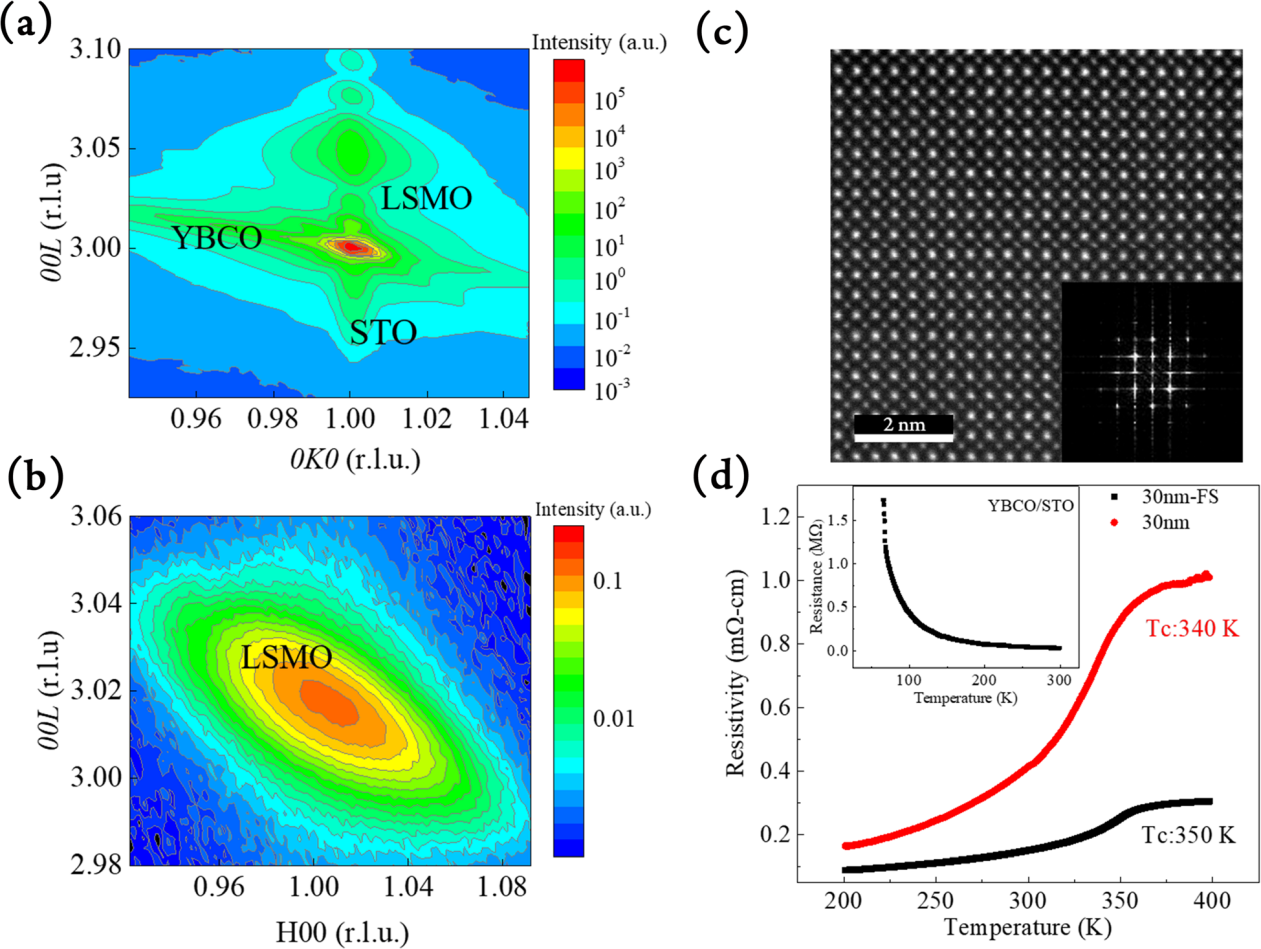
**a** Reciprocal space mapping (RSM) around as-grown LSMO (103) and **b** RSM around freestanding LSMO (103). **c** Plan view high-angle annular dark-field (HAADF) image of freestanding LSMO. The inset is the FFT pattern. **d** Temperature dependence of resistivity of LSMO before and after freestanding. The inset represents the transport behavior of as-grown single insulating YBCO sacrificial layer

In order to reveal the strain states and lattice variation of epitaxial LSMO before and after freestanding, XRD reciprocal space mapping (RSM) was conducted. The RSM around STO (103) plane is shown in Fig. 3a. For the as-grown sample, the in-plane lattice of LSMO film was fully strained by STO substrate, showing nearly the same in-plane lattice constants as STO single crystal. In this manner, the out-of-plane lattice of LSMO is shortened due to epitaxial constrain, which is consistent with the XRD normal scan. After etching, the RSM of freestanding LSMO exhibits a strain-free feature, as shown in Fig. 3b. The lattice constants of LSMO extracted from RSM data suggest a pseudo-cubic structure with lattice constants ~ 3.88 Å, which are the same as bulk values. This result indicates that the freestanding thin films possessing no strain after freestanding process, and there is no chemical bonding between transferred layer and carrier substrate. To further investigate more details of structural properties of freestanding LSMO, scanning transmission electron microscope (STEM) was employed. The high-angle annular dark-field (HAADF) image of freestanding LSMO, as presented in Fig. 3c, shows a defect-free and pseudo-cubic LSMO films. The corresponding fast Fourier transform (FFT) pattern of LSMO shown in the inset of Fig. 3c reveals the 4-fold and cubic in-plane lattice, which is nicely consistent with XRD results. Based on the above results, we have presented high-quality single-crystalline (001)-oriented LSMO films with the totally strain-free and no degradation features can be successfully transferred onto Si substrate.

We then turned to study the transport behaviors of LSMO films before and after freestanding. LSMO is a ferromagnetic material with Curie temperature (T_C_) as well as metal-insulator transition (MIT) at 369 K in bulk [19]. The transition temperature of LSMO is dominated by the double exchange interaction between Mn^3+^-O-Mn^4+^ [20]. In the LSMO/YBCO/STO heterostructure, the tensile strain will elongate the Mn-O-Mn bonds and cause a decrease in T_C_ [21]. As a result, there is an obvious decrease of T_C_ in as-grown LSMO. As shown in Fig. 3d, the T_C_ of as-grown LSMO is around 338 K. After removing YBCO bottom layer, as evidenced by XRD, there is no strain observed on the freestanding LSMO layer and the measured T_C_ is increased to 351 K. The inset of Fig. 3d represents the temperature dependence of resistivity of pure YBCO sacrificial layer on STO. In this study, a very thin and oxygen-deficient YBCO layer exhibiting insulating state was used. The adoption of insulating YBCO ensures the transport behavior of the as-grown sample was dominated by LSMO and thus precluding the metallic conduction caused by the YBCO layer. Moreover, a series of LSMO with various thickness have been fabricated to compare the T_C_ for as-grown and freestanding films. As shown in Supplementary Information Fig. S2, the transport behaviors of LSMO films with 16, 30, and 60 nm in thickness were measured before and after freestanding process. Generally speaking, the T_C_ of LSMO films are higher than those of as-grown samples, which is due to the absence of substrate clamping in freestanding LSMO layers after etching the YBCO sacrificial layers.

The etching process of YBCO could possibly be dominated by copper oxide, which can be treated as a weak base. The copper oxide constitution could rapidly react with HCl, forming soluble copper (II) chloride and water. The formula for the reaction of HCl with copper oxide can be denoted as:

$$ \mathrm{CuO}+2\mathrm{HCl}\to C{\mathrm{u}}^{2+}+2{\mathrm{Cl}}^{-}+2{\mathrm{H}}_2\mathrm{O} $$

**Fig. 4:**
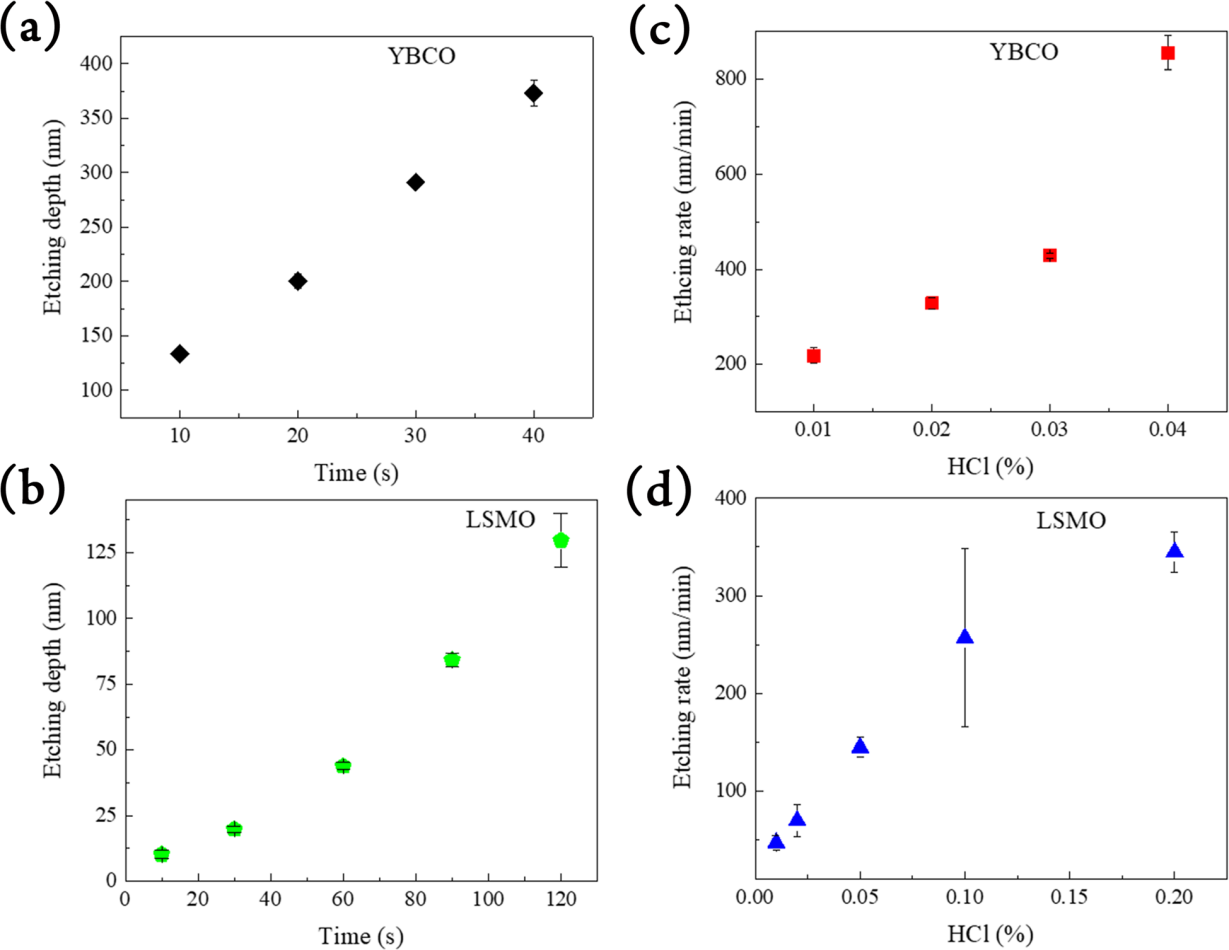
Etching depth versus time for **a** YBCO and **b** LSMO, respectively. HCl concentration-dependent etching rate of **c** YBCO and **d** LSMO

Using the same etching solution, the etching depth of YBCO can achieve over 120 nm in 10 s while LSMO would take more than 120 s to reach the same etching level, as shown in Fig. 4a, b, respectively. The etching rate of YBCO versus the concentration of HCl is further plotted in Fig. 4c. Compared to LSMO, as shown in Fig. 4d, the etching rate of YBCO is at least five times faster than that of LSMO, under the same concentration of HCl. Such a huge disparity can be attributed to the activity of chloride ion in HCl, where the activity of chloride ions will be strongly reduced while reacting with metal oxide that are in higher valences [22]. The manganese in LSMO is mixed-valence states composed of Mn^+3^ and Mn^+4^, while the valence state of copper in YBCO shifts from Cu^+1^ to Cu^+2^ during reaction [23]. As a result, the etching process of YBCO is much faster than LSMO. Habbache et al. [24] have reported that copper oxide will be etched in HCl significantly faster than other acids due to the aggressive character of the chloride anion. As a result, the use of YBCO as a sacrificial layer provides a faster approach which not only shortens the required re-action period, but also avoids the degradation of desired materials.

**Fig. 5:**
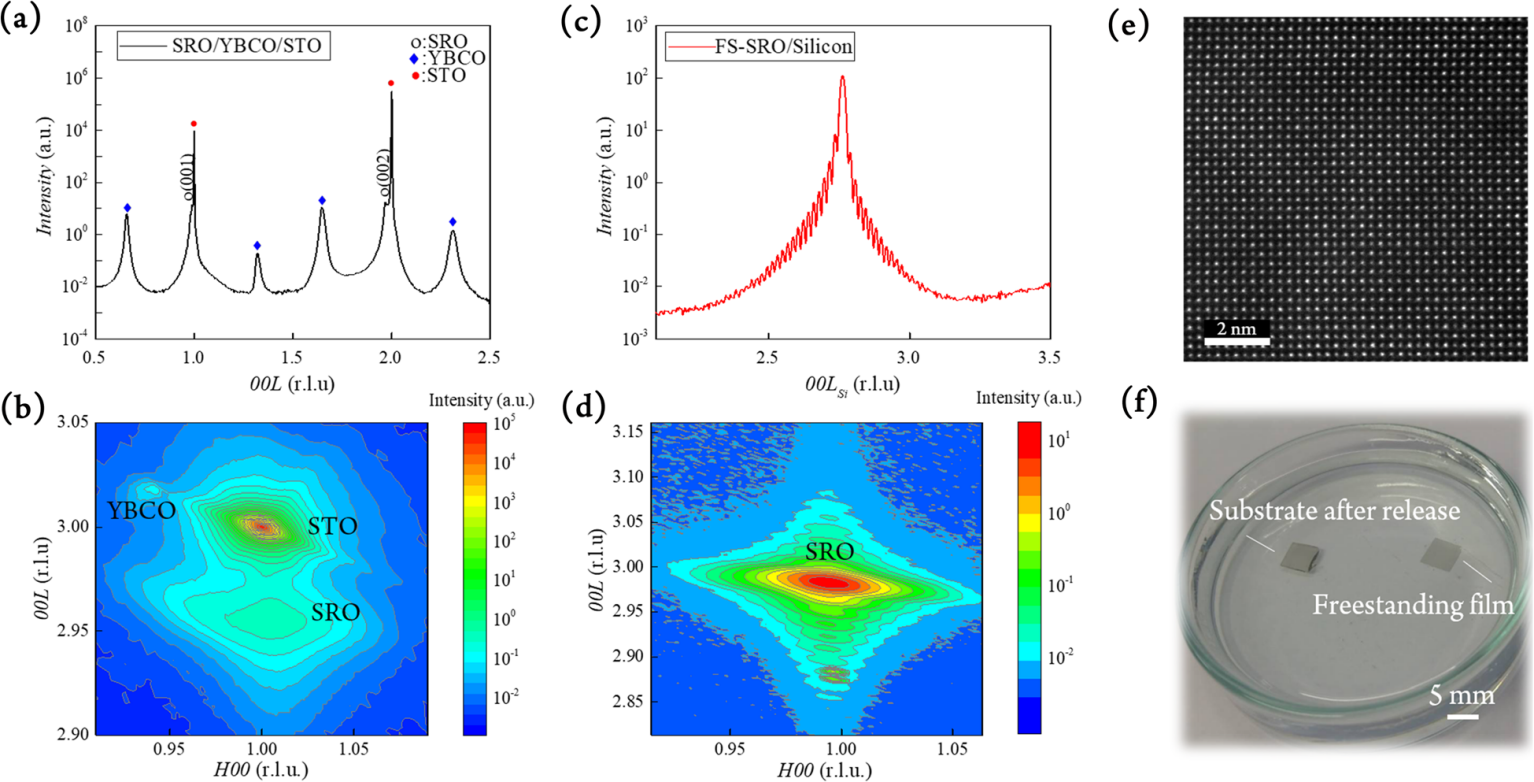
**a** XRD normal scan and **b** RSM of as-grown SRO heterostructure. **c** XRD normal scan and **d** RSM of freestanding SRO. **e** Plane view STEM HAADF image of freestanding SRO. **f** Photograph of freestanding SRO film and substrate after etching

To prove the versatile capability of using YBCO as a sacrificial layer, a similar procedure has been adopted to obtain SrRuO_3_ freestanding thin films. The corresponding surface morphology of SRO, as-grown and freestanding, are shown in Supplementary Information Fig. S3, evidencing a smooth surface without observable damages after freestanding. The XRD normal scan of SRO/YBCO/STO structure shown in Fig. 5a reveals the (001)-oriented pseudo-cubic crystalline without any secondary phase. The calculated *d*-spacing of as-grown SRO on STO is 3.96 Å, which is larger than the bulk value (3.93 Å), indicating a compressive strain applied on the SRO layer. The epitaxial constrain of SRO was revealed by RSM. As shown in Fig. 5b, SRO film was strained by STO, and thus, an elongated *c*-axis is observed. After the YBCO layer was removed by HCl solution, the XRD normal scan and RSM showed a strain-free SRO layer with lattice constant equals to 3.93 Å, as shown in Fig. 5c, d, respectively. The FWHM of as-grown and freestanding SRO (002) are 0.91° and 0.42°, respectively, showing a better quality of crystallinity after etching. Besides, the HAADF image, as shown in Fig. 5e, shows a defect-free and pseudo-cubic in-plane lattice of SRO freestanding membrane. The electrical transport behavior once again presents that substrate clamping effect on SRO films was removed after freestanding. In Supplementary Information Fig. S4, various thicknesses of SRO films were fabricated and compared. The freestanding SRO thin films always show higher T_C_ than as-grown ones due to the released epitaxial strains. Figure 5f shows a real photograph of freestanding thin films, in which the freestanding layer remain intact after freestanding, which also suggest large-scale freestanding systems is applicable. The successful demonstration of freestanding SRO suggests that using YBCO as a sacrificial layer is a universal method to acquire freestanding and single-crystalline functional oxides.

## Conclusion

In summary, we have established a fast route for fabricating high-quality single-crystalline freestanding thin films by using YBCO as a sacrificial layer. Through the modeling system, LSMO, as an example, high-quality and epitaxial LSMO freestanding thin films can be arbitrarily transferred onto silicon or any other existing substrates or devices. The XRD and STEM have evidenced that the epitaxial structures are properly maintained, while substrate clamping is completely removed after freestanding process. The freestanding features are also supported by the electrical transport measurements, by which the Curie temperature of the freestanding systems are found significantly higher due to the absent epitaxial constrain. The use of YBCO as a sacrificial layer has been verified that can be etched much faster than the conventional LSMO and SAO sacrificial layers, while doing unnoticeable damages to the targeted materials. With a similar approach adopted to fabricated freestanding SRO thin film, we have demonstrated using YBCO a generally versatile method to acquire freestanding oxide thin films. Our discovery offers a new perspective of utilities of functional oxide to the integration of silicon-based devices and flexible electronics.

## Supplementary information

**Additional file 1: Figure S1.** (a) Full width at half-maximum (FWHM) from the rocking curve around as grown LSMO (002) and (b) freestanding LSMO. **Figure S2.** Transport behaviors of LSMO films with (a) 16 nm, (b) 30 nm, and (c) 60 nm in thickness were measured before and after freestanding process. **Figure S3.** Surface morphology of (a) as grown SRO and (b) freestanding SRO. **Figure S4.** Transport behaviors of SRO films with (a) 15 nm, (b) 40 nm, (c) 65 nm, and (d) 130 nm in thickness were measured before and after freestanding process.

## Data Availability

The datasets supporting the conclusions of the article are included within the article.

## Abbreviations

YBCO: YBa_2_Cu_3_O_7_ Yttrium barium copper oxide
SAO: Sr_3_Al_2_O_6_ tri-strontium aluminates
PZT: Pb(Zr_0.2_Ti_0.8_)O_3_ lead zirconate titanate
STO: SrTiO_3_ strontium titanate
LSMO: La_*x*_Sr_1-*x*_MnO_3_ lanthanum strontium manganite
SRO: SrRuO_3_ strontium ruthenate
PMMA: Poly(methyl methacrylate)
STEM: Scanning transmission electron microscopy
HAADF: High-angle annular dark-field
PPMS: Physical property measurement system
DI water: Deionized water
XRD: X-ray diffraction
FWHM: Full width at half-maximum
RSM: Reciprocal space mapping
FFT: Fast Fourier transform
T_C_: Curie temperature
MIT: Metal-insulator transition
