# Supplementary material for: A Fast Route Towards Freestanding Single-Crystalline Oxide Thin Films by Using YBa2Cu3O7-x as a Sacrificial Layer
Source: Nanoscale Res Lett. 2020 Aug 28;15:172. doi: 10.1186/s11671-020-03402-0 (PMC7455685; doi:10.1186/s11671-020-03402-0)
Supplement: Supplementary file 1 — Additional file 1: Figure S1. (a) Full width at half-maximum (FWHM) from the rocking curve around as grown LSMO (002) and (b) freestanding LSMO. Figure S2. Transport behaviors of LSMO films with (a) 16 nm, (b) 30 nm, and (c) 60 nm in thickness were measured before and after freestanding process. Figure S3. Surface morphology of (a) as grown SRO and (b) freestanding SRO. Figure S4. Transport behaviors of SRO films with (a) 15 nm, (b) 40 nm, (c) 65 nm, and (d) 130 nm in thickness were measured before and after freestanding process. [file 11671_2020_3402_MOESM1_ESM.pdf]

Supporting Information

A fast route towards freestanding single crystalline oxide thin films by using $YBa_2Cu_3O_{7-x}$ as a sacrificial layer

Yao-Wen Chang[1†], Ping-Chun Wu[1†], Jhih-Bang Yi[1], Yu-Chen Liu[1], Yi Chou[2], Yi-Chia Chou[2], and Jan-Chi Yang[1,3*]

[1] Department of Physics, National Cheng Kung University, Tainan 70101, Taiwan

[2] Department of Electrophysics, National Chiao Tung University, Hsinchu 30010, Taiwan

[3] Center for Quantum Frontiers of Research & Technology (QFort), National Cheng Kung University, Tainan, 70101, Taiwan

Corresponding Author E-mail

* janchiyang@phys.ncku.edu.tw

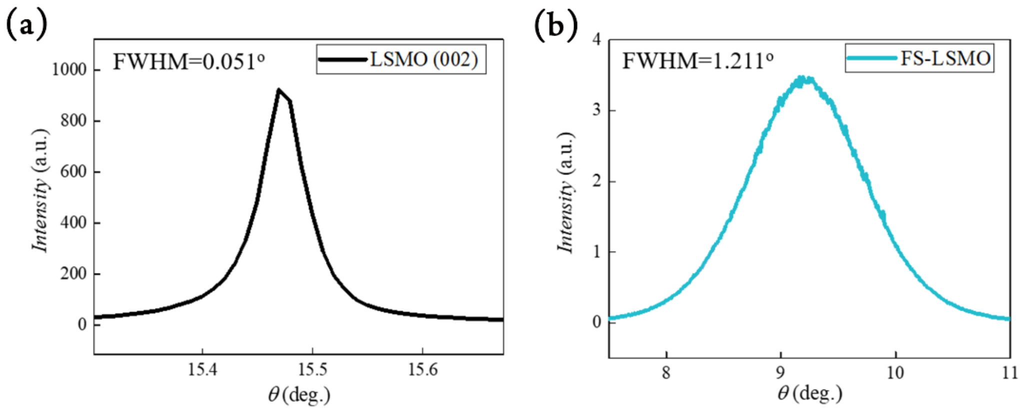

Figure S1. (a) Full width at half-maximum (FWHM) from the rocking curve around as grown LSMO (002) and (b) freestanding LSMO.

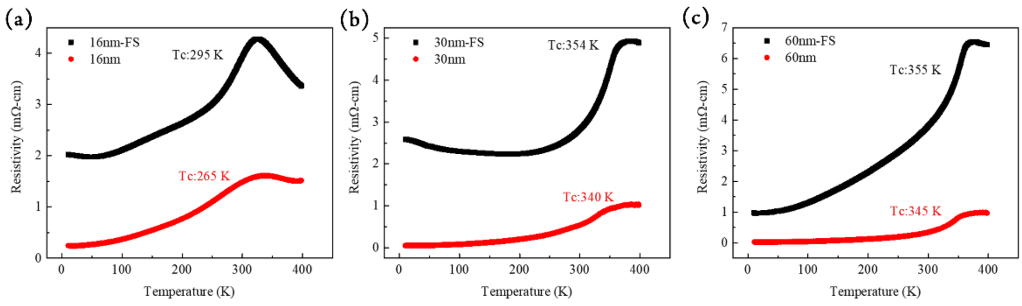

Figure S2. Transport behaviors of LSMO films with (a) 16 nm, (b) 30 nm, and (c) 60 nm in thickness were measured before and after freestanding process.

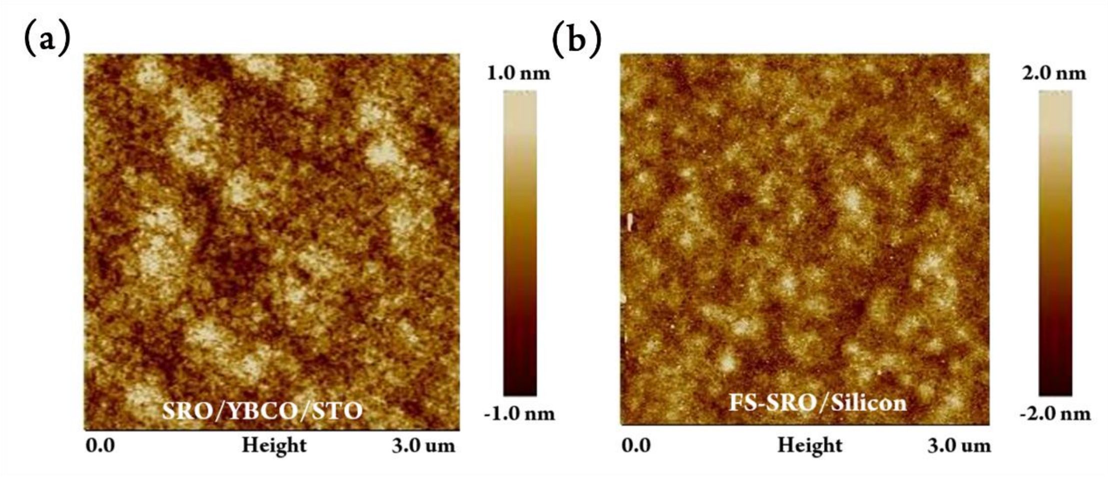

Figure S3. Surface morphology of (a) as grown SRO and (b) freestanding SRO.

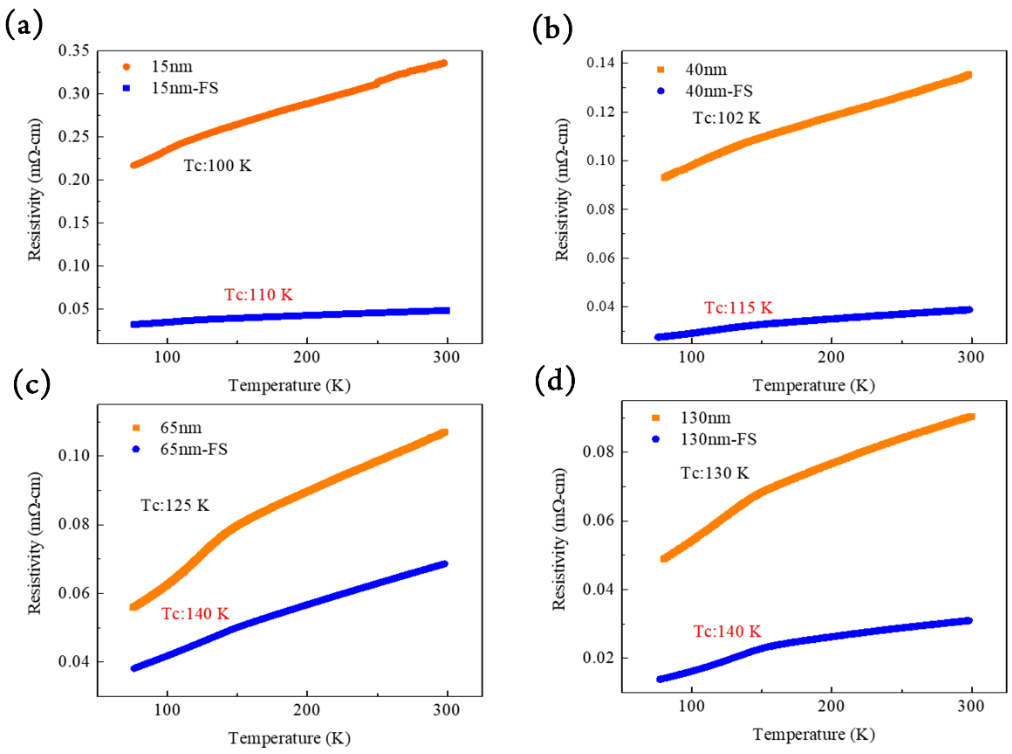

Figure S4. Transport behaviors of SRO films with (a) 15 nm, (b) 40 nm, (c) 65 nm, and (d) 130 nm in thickness were measured before and after freestanding process.
